# Health Care Access and Use Among Adults Experiencing Homelessness

**DOI:** 10.1001/jamahealthforum.2025.0820

**Published:** 2025-05-23

**Authors:** Jessica D. Fields, Ryan D. Assaf, Kim Hanh Nguyen, Corbin C. Platamone, J. Margo Pottebaum, Jesica Giannola, Margot B. Kushel

**Affiliations:** 1 Benioff Homelessness and Housing Initiative, Division of Health Equity and Society, Department of Medicine, University of California, San Francisco; 2 University of California, Berkeley–University of California, San Francisco Joint Medical Program, University of California, Berkeley; 3 School of Medicine, University of California, San Francisco; 4 Lived Expertise Advisory Board, Benioff Homelessness and Housing Initiative, Division of Health Equity and Society, Department of Medicine, University of California, San Francisco

## Abstract

**Importance:**

Demographic and policy changes have occurred since the last large, representative study of homeless adults in the 1990s, which may affect health care access and use.

**Objective:**

To describe the prevalence of poor health care access and short-term health care and use the Gelberg-Andersen Behavioral Model for Vulnerable Populations to assess the association between changes in homelessness with health care access and short-term care use.

**Design, Setting, and Participants:**

This representative survey of adults experiencing homelessness in California from October 2021 to November 2022 used multistage, venue-based, and respondent-driven sampling. Data were analyzed from May 2023 to December 2024.

**Exposures:**

Shelter status (predisposing vulnerable), insurance (enabling), impairment with activities of daily living (ADL; need), and illicit substance use during the previous 6 months (need).

**Main Outcomes and Measures:**

The study assessed self-reported no prior-year ambulatory care use and prior 6-month unmet health care need, unmet medication need, emergency department (ED) use, and hospitalization. Population prevalence estimates with Wald 95% CIs and multivariable Poisson regressions were calculated to compute prevalence ratios (PRs).

**Results:**

Thirty-two hundred adults completed the survey (mean age, 46.1 [95% CI, 45.3-46.9] years; 1965 cisgender men [67.2%], 1148 cisgender women [31.2%], and 57 transgender and gender queer individuals [1.6%]), of whom 2016 (77.6%) were unsheltered, 2609 (82.6%) were insured, 1056 (34.4%) had an ADL impairment, and 911 (37.1%) reported illicit substance use 3 or more times a week. A total of 1121 (39.1%) reported no ambulatory care use; 765 (24.3%) reported an unmet health care need and 714 (23.3%) an unmet medication need; 1252 (38.9%) used the ED; and 668 (22.0%) were hospitalized. Lack of ambulatory care use (PR, 1.71; 95% CI, 1.51-1.94) and unmet health care needs (PR, 1.19; 95% CI, 1.02-1.40) were more prevalent for those who were unsheltered. Lack of ambulatory care use (PR, 0.63; 95% CI, 0.57-0.70) and unmet health care needs (PR, 0.80; 95% CI, 0.67-0.95) were less prevalent for those with insurance. Unmet health care needs (PR, 2.13; 95% CI, 1.79-2.55), ED use (PR, 1.15; 95% CI, 1.02-1.30), and hospitalization (PR, 1.74; 95% CI, 1.40-2.17) were more prevalent for those with an ADL impairment. Lack of ambulatory care use (PR, 1.46; 95% CI, 1.19-1.79) and unmet health care needs (PR, 1.30; 95% CI, 1.08-1.55) were more prevalent for those who used illicit substances 3 or more times a week.

**Conclusions and Relevance:**

This cross-sectional study found that adults experiencing homelessness reported poor access to ambulatory care and a high prevalence of short-term care use, despite high rates of insurance. Changes in homelessness during the past 30 years were associated with worsened health care access and use.

## Key Points

**Question:**

What is the prevalence of and factors associated with health care access and use among adults experiencing homelessness in California?

**Findings:**

In this cross-sectional study of 3200 adults experiencing homelessness in California, there was low ambulatory care use, high unmet needs for health care and medication, and high short-term care use, but most participants had insurance. Being unsheltered, having impairment with activities of daily living, and illicit substance use were associated with poor health care access; being insured was associated with having access.

**Meaning:**

The study results suggest that people experiencing homelessness have limited access to health care and high short-term care use despite high rates of insurance.

## Teaser

This cross-sectional study examines the prevalence of poor health care access and short-term health care and use among people experiencing homelessness in California.

## Introduction

Poor health is associated with an increased risk of homelessness, and homelessness is detrimental to health.^1,2,3,4,5^ People experiencing homelessness face barriers to accessing and using ambulatory health care, including cost, competing priorities to meet basic needs, transportation issues, insurance challenges, and experiences of stigma and discrimination in health care settings.^6^ With poor health and access to care, they experience higher rates of emergency department (ED) use and hospitalization than the general population.^6,7,8^

To our knowledge, the last large representative study of people experiencing homelessness in the US was conducted from 1995 to 1996 (the National Survey of Homeless Assistance Providers and Clients [NSHAPC]).^9^ Since then, there have been numerous changes in who experiences homelessness and how they experience it, including the aging of the population and an increase in unsheltered homelessness^10,11,12^; changes in drug use patterns, including a shift toward methamphetamine use and an increase in drug-related overdoses^13,14^; and the reform and expansion of Medicaid and health care services.^1,15,16,17^ The COVID-19 pandemic accelerated the adoptions of tailored homeless health services.

In 2023, more than 650 000 people experienced homelessness on any given night in the US; 28% of the homeless population of the US and 49% of its unsheltered population reside in California.^11^ This study used a large, representative sample of adults experiencing homelessness in California to describe the prevalence of poor health care access and short-term health care use overall and by predisposing (traditional and vulnerable), enabling, and need factors from the Gelberg-Andersen Behavioral Model for Vulnerable Populations^18^ and the association of these factors with poor health care access and having an ED visit or hospitalization during the previous 6 months.

## Methods

### Study Overview

The California Statewide Study of People Experiencing Homelessness (CASPEH) was a representative mixed-methods study of people experiencing homelessness in California that was conducted between October 2021 and November 2022.^19,20^ We collaborated with 3 community advisory boards. The University of California, San Francisco institutional review board approved all study procedures. This study followed the Strengthening the Reporting of Observational Studies in Epidemiology (STROBE) reporting guidelines.

### Sampling Overview

We used a multistage, venue-based sampling design with randomization at 3 levels: counties, venues, and individuals. We divided California into 8 regions and randomly sampled 1 county from each region while maintaining the demographic distribution of the state’s general and homeless populations. After constructing a list of venues where people experiencing homelessness gather, we sampled venues using probability proportional to size. Within venues, we randomly sampled participants. To supplement our venue-based sampling, we used respondent-driven sampling, a social network–based sampling method, to reach populations we would otherwise have missed.^19^

### Eligibility

Participants were eligible if they were 18 years or older, experiencing homelessness according to Homeless Emergency Assistance and Rapid Transition to Housing Act criteria, able to provide informed consent using a teach-back method, and did not have an active COVID-19 infection.^21,22^ The study team administered a 45- to 60-minute survey in English or Spanish, with interpreters for other languages. Participants received a $35 grocery or $30 cash gift card.

### Theoretical Model

We used the Gelberg-Andersen model to guide study conceptualization, variable selection, and model building.^18^ This model conceptualizes health care utilization as a function of individuals’ predisposition to use health services (predisposing factors), factors that enable or impede use (enabling factors), and factors that determine health care need (need factors).

### Measures

#### Dependent Variables

We assessed 5 dependent variables: 3 self-reported measures of poor health care access (no ambulatory care use during the prior year, unmet health care needs, and unmet medication needs during the prior 6 months) and 2 self-reported measures of short-term health care utilization (ED use and hospitalization for physical health problem during the prior 6 months).^23^ To assess access to non-ED ambulatory care, we asked participants to report how long it had been since they last saw a health care clinician in a non-ED setting. We assessed unmet needs for health care and medication by asking whether participants needed but were unable to get health care or prescribed medications during the previous 6 months. We assessed ED use and hospitalization by asking participants how many times they had gone to the ED or how many times they had been hospitalized overnight during the previous 6 months.

#### Independent Variables

To isolate changes in homelessness since the last representative study, we focused on 4 independent variables: shelter (predisposing traditional factor), health insurance (enabling), impairment in activities of daily living (ADL; need), and illicit substance use (need). We asked participants where they slept the most nights during the previous 6 months while experiencing homelessness and dichotomized shelter status as sheltered vs unsheltered. We assessed insurance coverage by asking participants if they were covered by any kind of health insurance. We used the 5-item Katz Activities of Daily Living scale (any vs none).^24^ We adapted the World Health Organization ASSIST measures to examine past 6-month frequency of illicit substance use for 3 drugs: methamphetamine or amphetamine, nonprescription opioids, and cocaine.^25^ We created a single variable for illicit substance use of the 3 drugs and categorized frequency of use as regular (daily or almost daily, 3 or more times a week), occasional (more than twice a month but fewer than 3 times a week, once or twice a month, or less than monthly), and no use.

#### Confounding and Modeling Strategy

Based on the Gelberg-Andersen model and prior literature, we selected variables that came from the previous level to control for confounding for each independent variable. Additionally, we created correlation matrices; we selected 1 when 2 variables were 0.50 or more correlated. We did not control for variables from the same level as these may have mediated the exposure-outcome association.

#### Control and Descriptive Variables

We categorized age as 18 to 24, 25 to 49, 50 to 64, and 65 years or older. We assessed gender (cisgender women, cisgender men, and transgender/gender nonconforming). To account for structural racism, we used race and ethnicity as a proxy.^26^ We asked participants to self-report their race and ethnicity and categorized participants as African American/Black if they stated African American/Black regardless of another race and ethnicity. We categorized other racial groups as American Indian/Alaskan Native, Asian/Pacific Islander, Hispanic/Latinx, White, multiracial and multiethnic, or another race. For modeling purposes only, we collapsed American Indian/Alaskan Native, Asian/Pacific Islander, and another race not listed as a single category. We dichotomized participants’ preferred language (English and not English). We categorized veteran status as active duty, reserves, or National Guard vs none. We assessed recent carceral experience (either jail or prison incarceration immediately before this episode, prison incarceration during the 6 months before this episode, or jail incarceration during this episode). We used a rural-urban classification scheme based on population size, density, and commuting patterns using geocode data of where the interview took place; we dichotomized urbanicity as urban and suburban or rural.^27^

### Statistical Analysis

Data were analyzed from May 2023 to December 2024. We used weighted data, which we derived in 4 steps: (1) the joint probability of selection given our 3-stage cluster design at the county, venue, and individual level; (2) nonresponse; (3) combined venue-based and respondent-driven samples; and (4) poststratification to the 2022 point-in-time counts in California. We calculated 2-sided 95% CIs using survey replicate weights.^19^

We calculated unweighted sample size and weighted proportions with respective 95% CIs overall and by poor health care access and health care utilization for the independent and confounding variables from the Gelberg-Andersen model. We calculated Rao-Scott likelihood ratio χ^2^ tests for weighted data to assess the bivariate association. We did not report bivariate estimates or perform statistical testing when the unweighted sample was less than 100.

We modeled shelter status, insurance coverage, ADL impairment, and illicit substance use with each poor health care access and health care utilization outcome separately. We conducted unadjusted and adjusted multivariable Poisson regression for weighted data with robust estimation.^28^ We reported prevalence ratios (PRs) and 95% CIs.

Missingness for each independent variable and each outcome ranged from 3% to 4%; missingness for substance use was 7%. Missingness in the modeling ranged from 4% to 8% except for the adjusted model for substance use (11%). We conducted analyses in Stata MP, version 17.0 (StataCorp). Statistical significance was set at α < .05.

## Results

Overall, 3865 people were approached, of whom 3042 (79%) participated. Of those eligible (n = 3104), 98% participated. We recruited 158 participants through respondent-driven sampling.

A total of 1543 participants in California (51.3%) were age 25 to 49 years; 1965 (67.2%) identified as cisgender men, and 732 (26.3%) identified as African American/Black, 691 (26.4%) as Hispanic/Latinx, and 1089 (27.9%) as White. A total of 2016 (77.6%) experienced unsheltered homelessness. A total of 2609 (82.6%) reported having insurance coverage, and 1056(34.4%) of people reported at least 1 difficulty with ADL. Of people experiencing homelessness in California, 911 (37.1%) reported regular illicit substance use and 366 (12.5%) reported occasional use (Table 1).

**Table 1.** Characteristics of the 3200 Participants^a^

Characteristic	Overall	
	Unweighted No.	Weighted % (95% CI)^b^^,^^c^
Total	3200	
Predisposing traditional factors		
Age, y		
18-24	216	5.2 (4.0-6.5)
25-49	1543	51.3 (48.5-54.0)
50-64	1187	36.8 (33.9-39.7)
≥65	253	6.7 (5.7-7.6)
Gender identity		
Cisgender men	1965	67.2 (65.2-69.3)
Cisgender women	1148	31.2 (29.1-33.2)
Transgender and gender queer	57	1.6 (1.0-2.1)
LGBTQ+ sexual orientation	339	10.0 (8.5-11.4)
Race and ethnicity^d^		
African American/Black	732	26.3 (22.8-29.7)
American Indian and Alaska Native	107	2.9 (2.4-3.4)
Asian and Pacific Islander	64	1.7 (1.1-2.3)
Hispanic/Latinx	691	26.4 (23.4-29.4)
Multiracial and multiethnic	441	14.3 (12.2-16.4)
White	1089	27.9 (25.4-30.5)
Another race not listed	15	0.5 (0.3-0.8)
No high school education	996	36.0 (33.2-38.7)
Worked for pay for ≥20 h/wk	237	5.4 (4.6-6.2)
Not born in the US	395	12.7 (11.0-14.4)
English language	3039	94.5 (93.4-95.7)
Veteran status: yes	174	5.8 (4.7-6.8)
Predisposing vulnerable factors		
Had recent jail or prison carceral experience	1083	39.7 (37.3-42.0)
Time experiencing homelessness during current episode		
1 y	1186	34.6 (31.9-37.3)
1-3 y	913	29.7 (27.0-32.3)
>3 y	1096	35.7 (33.5-38.0)
Place slept most often during the previous 6 mo: unsheltered^e^	2016	77.6 (76.6-78.7)
Enabling factors		
Urban dwelling	2993	95.5 (94.3-96.8)
Had insurance coverage	2609	82.6 (79.9-85.3)
Had a regular source of health care	1683	50.7 (46.7-54.7)
Need factors		
Had impairment in activities of daily living	1056	34.4 (32.5-35.3)
Frequency of any illicit substance use (previous 6 mo)		
Regular^f^	911	37.1 (32.9-41.3)
Occasional^g^	366	12.5 (10.6-14.5)
None	1708	50.3 (47.2-53.4)

Abbreviations: LGBTQ+, lesbian, gay, bisexual, queer, plus other sexual orientations; NA, not applicable.

^a^ Sample n may not add to 3200 because of missing data.

^b^ Weighted percentages were calculated in 4 steps: (1) joint probability for selection; (2) nonresponse; (3) combined venue-based and respondent-driven samples; and (4) poststratification to the 2022 point-in-time counts in California.

^c^ Wald 95% CIs were calculated using survey replicate weights.

^d^ African American/Black race was treated as the determining group to account for anti-Black racism and the disproportion of Black American individuals experiencing homelessness.

^e^ Unsheltered included outdoors, street, park, tent, place, or vehicle not designed for people to live in.

^f^ Regular use defined as 3 or more times per week.

^g^ Occasional use defined as more than 2 times per month, once or twice per month, or less than per month.

### Poor Health Care Access

Overall, 1121 people (39.1%) reported no ambulatory care use during the previous year. A total of 765 (24.3%) reported an unmet health care need, and 714 (23.3%) reported an unmet medication need during the previous 6 months (Table 2).

**Table 2.** Prevalence Estimates of Poor Health Care Access by Exposure and Confounding Variables From the Gelberg-Andersen Model

Characteristic	Unweighted No. (overall)^a^	No ambulatory care use during prior year		Unmet health care need		Unmet medication need	
		Weighted % (95% CI)^b^^,^^c^	*P* value^d^	Weighted % (95% CI)^b^^,^^c^	*P* value^d^	Weighted % (95% CI)^b^^,^^c^	*P* value^d^
Total	3200	39.1 (35.8-42.4)		24.3 (22.5-26.0)		23.3 (21.1-25.4)	NA
**Exposures/characteristics**							
Predisposing vulnerable factors							
Place slept most often during the previous 6 mo							
Sheltered^e^	1111	23.5 (20.9-26.1)	<.001	22.3 (19.4-25.3)	.11	23.7 (20.2-27.1)	.87
Unsheltered^f^	2016	43.8 (39.6-48.0)		25.0 (23.0-27.0)		23.3 (20.9-25.8)	
Enabling factors							
Insurance coverage							
Yes	2609	34.5 (31.4-37.6)	<.001	23.6 (21.7-25.4)	.12	24.1 (21.7-26.4)	.05
No	481	60.4 (54.8-66.0)		27.4 (22.5-32.3)		18.9 (14.6-23.3)	
Need factors							
Impairment with activities of daily living							
Yes	1056	32.8 (28.0-37.6)	<.001	36.9 (33.3-40.5)	<.001	35.4 (30.6-40.1)	<.001
No	2054	42.1 (38.7-45.5)		17.4 (15.2-19.5)		16.6 (14.6-18.5)	
Frequency of any illicit substance use (previous 6 mo)							
Regular^g^	911	50.6 (45.1-56.1)	<.001	27.0 (23.2-30.9)	.03	22.4 (18.5-26.3)	.78
Occasional^h^	366	37.5 (28.4-46.6)		25.4 (19.2-31.6)		25.2 (19.0-31.4)	
No use	1708	30.5 (26.1-34.8)		21.0 (18.7-23.4)		22.6 (18.7-26.4)	
**Confounding/control variables**							
Predisposing traditional factors							
Age, y							
18-24	216	39.2 (30.7-47.7)	.008	22.7 (16.6-28.9)	.86	14.3 (9.5-19.2)	.003
25-49	1543	42.8 (38.6-47.1)		24.7 (21.8-27.6)		23.5 (20.5-26.6)	
50-64	1187	34.5 (29.5-39.5)		24.3 (21.0-27.7)		25.3 (22.4-28.2)	
≥65	253	36.0 (28.3-43.8)		21.9 (17.4-26.4)		16.8 (12.3-21.2)	
Gender identity							
Cisgender men	1965	41.6 (37.5-45.7)	.001	22.1 (19.7-24.5)	.007	20.7 (18.1-23.2)	.001
Cisgender women	1148	34.0 (30.3-37.7)		27.8 (24.7-30.9)		28.5 (24.6-32.4)	
Transgender and gender queer^f^	57	NA		NA		NA	
Race and ethnicity^i^							
African American/Black	732	27.0 (22.9-31.0)	<.001	26.0 (21.8-30.3)	.001	27.3 (22.9-31.8)	.01
American Indian and Alaska Native	107	32.7 (26.0-39.5)		24.8 (18.8-30.9)		22.9 (17.3-28.6)	
Asian and Pacific Islander^f^	64	NA		NA		NA	
Hispanic/Latinx	691	41.9 (36.3-47.6)		17.9 (14.5-21.4)		18.9 (14.7-23.1)	
Multiracial and multiethnic	441	36.4 (27.2-45.6)		24.2 (19.1-29.3)		26.7 (21.2-32.3)	
White	1089	47.9 (44.1-51.7)		27.9 (24.5-31.4)		22.5 (19.5-25.5)	
Another race not listed^j^	15	NA		NA		NA	
Language							
English	3039	39.0 (35.6-42.4)	.69	24.6 (22.8-26.5)	.07	23.7 (21.5-25.9)	.02
Not English	161	41.1 (31.5-50.6)		17.9 (11.9-23.9)		15.6 (10.1-21.2)	
Veteran status							
Yes	174	44.2 (32.6-55.9)	.33	18.1 (9.6-26.5)	.15	16.2 (8.5-24.0)	.13
No	3001	38.8 (35.5-42.1)		24.6 (23.0-26.2)		23.6 (21.3-25.9)	
Predisposing vulnerable factors							
Recent jail or prison carceral experience							
Yes	1083	42.1 (37.1-47.2)	.05	26.1 (22.4-29.8)	.13	25.0 (21.1-28.9)	.26
No	2031	36.8 (33.3-40.3)		22.8 (20.7-24.9)		21.9 (18.8-25.0)	
Enabling factors							
Urbanicity							
Urban	2993	39.2 (35.8-42.7)	.44	24.4 (22.5-26.2)	.23	23.3 (21.1-25.6)	.37
Suburban or rural	207	37.1 (33.1-41.1)		22.5 (19.9-25.0)		21.2 (17.4-25.0)	

Abbreviation: NA, not applicable.

^a^ Sample n may not add to 3200 because of missing data.

^b^ Weighted percentages were calculated in 4 steps: (1) joint probability for selection; (2) nonresponse; (3) combined venue-based and respondent-driven samples; and (4) poststratification to the 2022 point-in-time counts in California.

^c^ Wald 95% CIs were calculated using survey replicate weights.

^d^ The Rao-Scott χ^2^ test was used for weighted data (unadjusted).

^e^ Unsheltered included outdoors, street, park, tent, place, or vehicle not designed for people to live in.

^f^ Race and ethnicity. African American/Black race was treated as the determining group to account for anti-Black racism and the disproportion of Black Americans experiencing homelessness.

^g^ Regular use defined as 3 or more times per week.

^h^ Occasional use defined as more than 2 times per month, once or twice per month, or less than per month.

^i^ Sheltered location included emergency shelter, shelter for people fleeing domestic violence, motel or hotel room paid for by the government during COVID-19, motel or hotel room paid for by friends or family, mental health or drug or alcohol treatment program, or a family member or friend’s residence.

^j^ Weighted percentages were not calculated for this subpopulation because the sample was fewer than 100 individuals.

#### Shelter Status During the Previous 6 Months (Predisposing Vulnerable Factor)

Among those who were unsheltered, 867 (43.8%) had no ambulatory care use during the prior year and 524 (25.0%) had an unmet health care need, and 474 (23.3%) had an unmet medication need during the previous 6 months (Table 2). After adjusting for predisposing traditional factors, the prevalence of no ambulatory care use during the prior year was 1.71 times that for people in unsheltered locations compared with those in sheltered locations (95% CI, 1.51-1.94); the prevalence of unmet health care needs was 1.19 times (95% CI, 1.02-1.40), and the prevalence of unmet medication needs was 1.05 times (95% CI, 0.88-1.25) (Table 3).

**Table 3.** Unadjusted and Adjusted Poisson Regression for Poor Health Care Access

Characteristic	Prevalence ratio (95% CI)^a^		
	No ambulatory care use during prior year	Unmet health care need	Unmet prescription medication needs
**Shelter status, previous 6 mo (predisposing vulnerable)**			
Unadjusted model^b^			
Sheltered location	1 [Reference]	1 [Reference]	1 [Reference]
Unsheltered	1.86 (1.63-2.13)	1.12 (0.97-1.29)	0.99 (0.83-1.17)
Fully adjusted model^c^			
Sheltered location	1 [Reference]	1 [Reference]	1 [Reference]
Unsheltered	1.71 (1.51-1.94)	1.19 (1.02-1.40)	1.05 (0.88-1.25)
**Insurance coverage (enabling)**			
Unadjusted model^b^			
No or do not know	1 [Reference]	1 [Reference]	1 [Reference]
Yes	0.57 (0.52-0.63)	0.86 (0.72-1.03)	1.27 (1.00-1.61)
Fully adjusted model^d^			
No or do not know	1 [Reference]	1 [Reference]	1 [Reference]
Yes	0.63 (0.57-0.70)	0.80 (0.67-0.95)	1.08 (0.85-1.38)
**Activities of daily living impairment (need)**			
Unadjusted model^b^			
No	1 [Reference]	1 [Reference]	1 [Reference]
Yes	0.78 (0.68-0.89)	2.13 (1.80-2.51)	2.14 (1.79-2.55)
Fully adjusted model^e^			
No	1 [Reference]	1 [Reference]	1 [Reference]
Yes	0.82 (0.71-0.95)	2.13 (1.79-2.55)	2.08 (1.70-2.54)
**Illicit substance use, last 6 mo (need)**			
Unadjusted model^b^			
No use	1 [Reference]	1 [Reference]	1 [Reference]
Occasional use	1.23 (0.98-1.55)	1.21 (0.91-1.60)	1.12 (0.81-1.55)
Regular use	1.66 (1.40-1.97)	1.28 (1.09-1.51)	0.99 (0.78-1.27)
Fully adjusted model^e^			
No use	1 [Reference]	1 [Reference]	1 [Reference]
Occasional use	1.16 (0.93-1.44)	1.22 (0.94-1.58)	1.11 (0.78-1.58)
Regular use	1.46 (1.19-1.79)	1.30 (1.08-1.55)	0.99 (0.76-1.28)

^a^ Weighted percentages were calculated in 4 steps: (1) joint probability for selection; (2) nonresponse; (3) combined venue-based and respondent-driven samples; and (4) poststratification to the 2022 point-in-time counts in California.

^b^ Unadjusted Poisson regression for weighted data with robust estimation.

^c^ Adjusted model controlling for age, gender, race and ethnicity, language, and veteran status (predisposing traditional factors).

^d^ Adjusted model controlling for age, gender, race and ethnicity, language, veteran status, carceral experience, and shelter status (predisposing traditional + predisposing vulnerable factors).

^e^ Adjusted model controlling for age, gender, race and ethnicity, language, veteran status, carceral experience, shelter status, urbanity, and insurance (predisposing traditional + predisposing vulnerable + enabling factors).

#### Insurance Coverage (Enabling Factor)

Among those who had insurance, 816 (34.5%) had no ambulatory care use during the prior year; 604 (23.6%) had an unmet health care need and 594 (24.1%) had an unmet medication need during the previous 6 months (Table 2). After adjusting for predisposing traditional and vulnerable factors, the prevalence of no ambulatory care use during the prior year was 0.63 times that for those with insurance compared with those without (95% CI, 0.57-0.70); the prevalence of unmet health care need was 0.80 times (95% CI, 0.67-0.95), and the prevalence of unmet medication need was 1.08 times (95% CI, 0.85-1.38) (Table 3).

#### ADL Impairment (Need Factor)

Among those with an ADL impairment, 311 (32.8%) had no ambulatory care use during the prior year; 385 (36.9%) had an unmet health care need and 362 (35.4%) had an unmet medication need during the previous 6 months (Table 2). After adjusting for predisposing traditional, predisposing vulnerable, and enabling factors, the prevalence of no ambulatory care use during the prior year was 0.82 times that for those with an ADL impairment compared with those without (95% CI, 0.71-0.95); the prevalence of unmet health care need was 2.13 times (95% CI, 1.79-2.55), and the prevalence of unmet medication need was 2.08 times (95% CI, 1.70-2.54) (Table 3).

#### Illicit Substance Use During the Previous 6 Months (Need Factor)

Among those who used illicit substances regularly, 448 (50.6%) had no ambulatory care use during the previous year; 258 (27.0%) had an unmet health care need and 224 (22.4%) had an unmet medication need during the previous 6 months (Table 2). After adjusting for predisposing traditional, predisposing vulnerable, and enabling factors, the prevalence of no ambulatory care use in the prior year was 1.46 times that for people who reported regular illicit substance use compared with those who did not (95% CI, 1.19-1.79); the prevalence of unmet health care need was 1.30 times (95% CI, 1.08-1.55), and the prevalence of unmet health care need was 0.99 for people who reported regular illicit substance use compared with those who did not (95% CI, 0.76-1.28) (Table 3).

### Short-Term Health Care Utilization

Overall, 1252 people (38.9%) experiencing homelessness in California used the ED. A total of 668 (22.0%) had an overnight hospitalization stay during the previous 6 months (Table 4).

**Table 4.** Prevalence Estimates of Short-Term Health Care Utilization During the Prior 6 Months by Exposures and Confounding Variables From the Gelberg-Andersen Model

Characteristic	Unweighted No. (overall)^a^	ED use during the prior 6 mo		Hospitalization during the prior 6 mo	
		Weighted % (95% CI)^b^^,^^c^	*P* value^d^	Weighted % (95% CI)^b^^,^^c^	*P* value^d^
Total	3200	38.9 (35.7-42.1)	NA	22.0 (19.9-24.1)	NA
**Exposures/characteristics**					
Predisposing vulnerable factors					
Place slept most often during the previous 6 mo					
Sheltered^e^	1111	40.0 (35.5-44.6)	.63	23.2 (19.8-26.7)	.50
Unsheltered^f^	2016	38.6 (34.7-42.5)		21.8 (19.1-24.4)	
Enabling factors					
Insurance coverage					
Yes	2609	41.3 (37.9-44.6)	<.001	23.9 (21.5-26.3)	.001
No	481	27.6 (21.2-34.0)		13.7 (9.3-18.2)	
Need factors					
Activities of daily living impairment					
Yes	1056	44.3 (39.4-49.3)	.001	31.5 (27.0-36.0)	<.001
No	2054	36.0 (32.7-39.4)		16.8 (14.4-19.3)	
Frequency of any illicit substance use (previous 6 mo)					
Regular^g^	911	38.3 (33.8-42.7)	.04	21.5 (17.1-26.0)	.98
Occasional^h^	366	47.3 (37.2-57.3)		20.9 (16.4-25.4)	
No use	1708	36.5 (32.5-40.5)		21.7 (17.6-25.7)	
**Confounding/control variables**					
Predisposing traditional factors					
Age, y					
18-24	216	34.3 (25.4-43.2)	.47	21.5 (11.7-31.2)	.10
25-49	1543	39.3 (35.6-42.9)		19.9 (16.7-23.2)	
50-64	1187	39.8 (35.0-44.6)		25.5 (22.1-28.9)	
≥65	253	34.5 (27.9-41.1)		18.9 (12.6-25.1)	
Gender identity					
Cisgender men	1965	35.5 (31.8-39.3)	<.001	21.4 (18.9-23.9)	.53
Cisgender women	1148	46.1 (41.0-51.1)		22.9 (18.8-26.9)	
Transgender and gender queer^i^	57	NA		NA	
Race and ethnicity^i^					
African American/Black	732	36.0 (30.9-41.1)	.27	28.6 (23.7-33.6)	<.001
American Indian and Alaska Native	107	36.9 (30.0-43.8)		15.6 (10.5-20.7)	
Asian and Pacific Islander^i^	64	NA		NA	
Hispanic/Latinx	691	35.8 (29.0-42.6)		19.0 (15.0-23.1)	
Multiracial and multiethnic	441	44.7 (32.8-56.7)		24.2 (19.8-28.5)	
White	1089	40.9 (37.6-44.2)		17.8 (15.7-19.8)	
Another race not listed^j^	15	NA		NA	
Language					
English	3039	39.8 (36.6-42.9)	.001	22.0 (19.6-24.3)	.93
Not English	161	23.4 (14.6-32.2)		22.4 (14.0-30.8)	
Veteran status					
Yes	174	37.5 (25.4 (49.6)	.80	18.0 (12.4-23.6)	.21
No	3001	38.9 (35.9-41.9)		22.2 (19.9-24.6)	
Predisposing vulnerable factors					
Recent jail or prison carceral experience					
Yes	1083	39.4 (34.9-43.9)	.86	22.4 (19.1-25.7)	.71
No	2031	38.8 (34.6-43.1)		21.5 (18.5-24.6)	
Enabling factors					
Urbanicity					
Urban	2993	38.7 (35.3-42.0)	.18	21.9 (19.7-24.1)	.23
Suburban or rural	207	43.4 (36.9-49.8)		24.1 (21.1-27.0)	

Abbreviations: ED, emergency department; NA, not applicable.

^a^ Sample n may not add to 3200 because of missing data.

^b^ Weighted percentages were calculated in 4 steps: (1) joint probability for selection; (2) nonresponse; (3) combined venue-based and respondent-driven samples; and (4) poststratification to the 2022 point-in-time counts in California.

^c^ 95% Wald CIs were calculated using survey replicate weights.

^d^ *P* value: the Rao-Scott χ^2^ test was used for weighted data (unadjusted).

^e^ Sheltered location included emergency shelter, shelter for people fleeing domestic violence, motel or hotel room paid for by the government during COVID-19, motel or hotel room paid for by friends or family, mental health or drug or alcohol treatment program, a family member or friend’s residence.

^f^ Unsheltered included outdoors, street, park, tent, place, or vehicle not designed for people to live in.

^g^ Regular use defined as 3 or more times per week.

^h^ Occasional use defined as more than 2 times per month, once or twice per month, or less than per month.

^i^ Race and ethnicity. African American/Black race was treated as the determining group to account for anti-Black racism and the disproportion of Black Americans experiencing homelessness.

^j^ Weighted percentages were not calculated for this subpopulation because the sample was fewer than 100 individuals in the unweighted n.

#### Shelter Status During the Previous 6 Months (Predisposing Vulnerable Factor)

Among those who were unsheltered, 785 (38.6%) used the ED (95% CI, 34.7%-42.5%) and 432 (21.8%) had an overnight hospitalization (95% CI, 19.1%-24.4%) during the previous 6 months (Table 4). After adjusting for predisposing traditional factors, the prevalence of ED use was 0.94 times that for people in unsheltered locations compared with those in sheltered locations (95% CI, 0.82-1.08); the prevalence of hospitalization was 1.00 times (95% CI, 0.83-1.20) (Table 5).

**Table 5.** Unadjusted and Adjusted Poisson Regression of Short-Term Health Care Utilization During the Previous 6 Months

Characteristic	Prevalence ratio (95% CI)^a^	
	Emergency department use during the prior 6 mo	Hospitalization during the prior 6 mo
**Shelter status, previous 6 mo (predisposing vulnerable)**		
Unadjusted model^b^		
Sheltered location	1 [Reference]	1 [Reference]
Unsheltered	0.97 (0.83-1.12)	0.94 (0.77-1.14)
Fully adjusted model^c^		
Sheltered location	1 [Reference]	1 [Reference]
Unsheltered	0.94 (0.82-1.08)	1.00 (0.83-1.20)
**Insurance coverage (enabling)**		
Unadjusted model^b^		
No or do not know	1 [Reference]	1 [Reference]
Yes	1.50 (1.18-1.90)	1.74 (1.26-2.41)
Fully adjusted model^d^		
No or do not know	1 [Reference]	1 [Reference]
Yes	1.48 (1.19-1.83)	1.76 (1.32-2.35)
**Activities of daily living impairment (need)**		
Unadjusted model^b^		
No	1 [Reference]	1 [Reference]
Yes	1.23 (1.10-1.38)	1.87 (1.52-2.32)
Fully adjusted model^e^		
No	1 [Reference]	1 [Reference]
Yes	1.15 (1.02-1.30)	1.74 (1.40-2.17)
**Illicit substance use, previous 6 mo (need)**		
Unadjusted model^b^		
No use	1 [Reference]	1 [Reference]
Occasional use	1.29 (1.08-1.56)	0.96 (0.70-1.34)
Regular use	1.05 (0.92-1.20)	0.99 (0.74-1.33)
Fully adjusted model^e^		
No use	1 [Reference]	1 [Reference]
Occasional use	1.33 (1.14-1.57)	0.98 (0.68-1.42)
Regular use	1.07 (0.91-1.25)	1.06 (0.78-1.44)

^a^ Weighted percentages were calculated in 4 steps: (1) joint probability for selection; (2) nonresponse; (3) combined venue-based and respondent-driven samples; and (4) poststratification to the 2022 point-in-time counts in California.

^b^ Unadjusted Poisson regression for weighted data with robust estimation.

^c^ Adjusted model controlling for age, gender, race and ethnicity, language, and veteran status (predisposing traditional factors).

^d^ Adjusted model controlling for age, gender, race and ethnicity, language, veteran status, carceral experience, and shelter status (predisposing traditional + predisposing vulnerable factors).

^e^ Adjusted model controlling for age, gender, race and ethnicity, language, veteran status, carceral experience, shelter status, urbanity, and insurance (predisposing traditional + predisposing vulnerable + enabling factors).

#### Insurance Coverage (Enabling Factor)

Among those with insurance coverage, 1099 (41.3%) used the ED and 597 (23.9%) had an overnight hospitalization during the previous 6 months (Table 4). After adjusting for predisposing traditional and vulnerable factors, the prevalence of ED use was 1.48 times that for those with insurance compared with those without insurance (95% CI, 1.19-1.83); the prevalence of hospitalization was 1.76 times (95% CI, 1.32-2.35) (Table 5).

#### ADL Impairment (Need Factor)

Among those with an ADL impairment, 335 (44.3%) had used the ED and 335 (31.5%) had an overnight hospitalization during the previous 6 months (Table 4). After adjusting for predisposing traditional, predisposing vulnerable, and enabling factors, the prevalence of ED use was 1.15 times that for those with an ADL impairment compared with those without (95% CI, 1.02-1.30); the prevalence of hospitalization was 1.74 times (95% CI, 1.40-2.17) (Table 5).

#### Illicit Substance Use During the Previous 6 Months (Need Factor)

Among those who used illicit substances regularly, 368 (38.3%) had used the ED and 178 (21.5%) had an overnight hospitalization during the previous 6 months (Table 4). After adjusting for predisposing traditional, predisposing vulnerable, and enabling factors, the prevalence of ED use was 1.07 times that for people who reported regular illicit substance use compared with those who did not report illicit substance use (95% CI, 0.91-1.25); the prevalence of hospitalization was 1.06 times (95% CI, 0.78-1.44) (Table 5).

## Discussion

In a representative cross-sectional study of adults experiencing homelessness in California, homeless adults had poorer access to health care and higher short-term care use than those in the last large representative study of homelessness conducted in the 1990s.^7^ Despite important positive changes in health care for this population since then, including Medicaid expansion and expanded homelessness-tailored health care services, changes in who and how people experience homelessness contributed to poorer outcomes. A significantly higher proportion of people reported having health insurance in our study compared with NSHAPC; having insurance was associated with better access measures. Yet, since the 1990s, there has been a large rise in unsheltered homelessness, the population has aged (with a corresponding increase in people experiencing ADL limitations), and there have been shifts in drug use toward methamphetamine use and a higher risk of overdose.^10,11,13,15,16^ We found that being unsheltered, having ADL limitations, and using illicit drugs were associated with poor access to care and high use of short-term care. These changes diminished the potential improvements from increased insurance access and other health system changes.

Since California expanded Medicaid in 2014, most California adults who experience homelessness qualify for insurance.^29^ We found a higher proportion of insured individuals (81.6%) compared with NSHAPC (44.4%).^7^ Having health insurance was associated with a lower prevalence of not having received ambulatory care and of reporting an unmet need for health care. Having insurance was associated with higher use of the ED and inpatient hospitalization, as hospitals may obtain Medicaid for people.

Homelessness interferes with receiving health care through various mechanisms, including creating competing needs, increasing barriers to transportation and communication, and heightening stigma and discrimination.^30,31^ Recent expansions of health services tailored to people experiencing homelessness, such as shelter-based clinics and street outreach, may have been associated with improved access, as were innovations during the COVID-19 pandemic.^32^ However, Medicaid expansion and tailored services did not overcome the negative associations of other factors, such as the rise in unsheltered homelessness and the aging population.^10,11,12,20^

Since NSHAPC, unsheltered homelessness has increased markedly.^11,20^ People experiencing unsheltered homelessness face additional barriers to receiving health care beyond those encountered by sheltered adults, including the lack of safe places to store belongings while seeking care, limited ability to receive written appointment reminders or use mobile phones, and competing risks and stigma.^33^ Unsheltered homelessness was associated with lower health care access, as demonstrated by a lower prevalence of having a non-ED visit during the prior year and higher prevalence of unmet needs for health care, but not with differences in short-term health care use. The lack of association with short-term care may be associated with unmeasured confounders or to effect-cause, as hospitals may refer people to shelters following hospital visits.

The homeless population has aged.^10,12^ Nearly half of single homeless adults are 50 years and older, and they exhibit the health status of those 20 years older in the general population. This population includes a high prevalence of ADL impairments.^4,20^ Having an ADL impairment was associated with a lower prevalence of not having had an ambulatory care visit but a higher prevalence of reporting unmet needs for health care and medications, having an ED visit, and an inpatient hospitalization. ADL impairments increase a person’s need for health care services. While people with ADL impairments were more likely to have seen a clinician during the prior year, it was not enough to meet their needs.

Using illicit drugs was associated with a higher prevalence of not having had an ambulatory care visit and reporting an unmet need for health care, as well as a higher likelihood of reporting an ED visit. Regular illicit drug use was associated with lower use of ambulatory care. The associations of illicit drug use with social functioning can hinder the advanced planning needed for scheduled appointments. Additionally, stigma and discrimination from health care clinicians toward people who use drugs can further limit access to and use of ambulatory care.

### Limitations

Our study had limitations. Because the data were cross-sectional, we could not assess temporality or causality. The findings may not be generalizable to states that did not expand Medicaid or to those where people experiencing homelessness are guaranteed shelter. Although we relied on self-reported health care utilization, prior work has shown that people experiencing homelessness are as reliable in self-reporting as the general population, and we used 6-month time scales to improve recall.^34^

The sample frame of NSHAPC was restricted to those receiving homeless services, whereas our study included all adults who experienced homelessness regardless of service interaction. People who interact with homeless services may have better access to health services. This difference may account for some of the contrasts. Our study differed from studies of homelessness that included only those who use health care, which may mischaracterize the prevalence of health care utilization among all adults who experience homelessness, or those that recruited convenience samples from people staying in homeless shelters.^8,35,36^ Other studies were based outside the US or were small and specific to individual US cities, lacking generalizability.^37,38,39^

## Conclusions

In this cross-sectional, representative study of adults experiencing homelessness in California, we found poor access to health care and high rates of short-term health care utilization that reflected how homelessness is negatively associated with health care access. Predisposing contextual and need factors, including being unsheltered, using illicit drugs, and having functional impairments, were associated with reduced access to care and increased use of short-term care. These findings underscore the necessity for health care and housing interventions that are tailored to the needs of people experiencing homelessness.

## Notes

Supplement. Data sharing statement
