# Supplementary material for: Health Care Access and Use Among Adults Experiencing Homelessness
Source: JAMA Health Forum. 2025 May 23;6(5):e250820. doi: 10.1001/jamahealthforum.2025.0820 (PMC12102704; doi:10.1001/jamahealthforum.2025.0820)
Supplement: Supplement. — Data sharing statement [file jamahealthforum-e250820-s001.pdf]

Data Sharing Statement

Fields. Health Care Access and Use Among Adults Experiencing Homelessness. *JAMA Health Forum*. Published May 23, 2025. doi:10.1001/jamahealthforum.2025.0820

Data
Data available: No

Additional Information
Explanation for why data not available: privacy
